# Task engagement and mental workload involved in variation and repetition of a motor skill

**DOI:** 10.1038/s41598-017-15343-3

**Published:** 2017-11-07

**Authors:** Natália Lelis-Torres, Herbert Ugrinowitsch, Tércio Apolinário-Souza, Rodolfo N. Benda, Guilherme M. Lage

**Affiliations:** School of Physical Education, Physiotherapy and Occupational Therapy, Universidade Federal de Minas Gerais, Av. Presidente Carlos Luz, 6627, Pampulha, 31270-901 Belo Horizonte Brazil

## Abstract

Explanatory hypotheses proposed in behavioral studies assumed that less repetitive practice schedules, such as random practice, seem to demand greater cognitive effort than more repetitive types of practice organization, such as constant. All of these hypotheses emphasize the enhanced demand to memory processes promoted by less repetitive practice schedules. In the present study, we investigated the cognitive effort involved in random and constant practice schedules with an electrophysiological approach. Twenty-one male participants practiced a sequential key-pressing task with two goals: learning the relative timing dimension and learning the absolute timing dimension. Sixty trials were performed in a constant practice schedule (only one absolute timing goal), and sixty trials were performed in random order (three absolute timing goals). Two electroencephalography based measures of cognitive states were used: (a) task engagement (sensory processing and attention resources) and (b) mental workload (working memory load). The results showed that random practice induced greater cognitive effort than constant practice when task engagement was analyzed. Throughout practice, both task engagement and mental workload decreased more in the constant practice condition than in the random practice condition. The increased demand for sensory processing observed in random practice opens a new exciting field of study in practice organization.

## Introduction

While constant practice consists of practicing only one skill during a practice session (A-A-A-A-A-A-A-A-A), variable practice involves the practice of two or more skills or variations of a same skill^1^. Variable practice with a non-systematic order of skill execution (A-C-B-C-A-B-A-B-C), or variations of a same skill (A-A2-A1-A2-A1-A-A1-A2-A), is defined as random (or interleaved) practice. Conversely, variable practice with consecutive execution of a given skill before the execution of the block of another skill (A-A-A-B-B-B-C-C-C), or before the execution of the block of another variation of the same skill (A-A-A-A1-A1-A1-A2-A2-A2), is called blocked practice. Repetition and variation in motor practice produces different levels of cognitive effort and learning^2^. Less repetitive practice schedules, such as random practice, require greater cognitive effort than more repetitive types of practice organization, such as constant and blocked. Consequently, less repetitive practice schedules promote better learning^3^.

The assumption that less repetitive practice schedules promote greater cognitive effort has been investigated almost solely at the behavioral level. The variability of practice hypothesis claims that variable practice produces a stronger memory schema than constant practice to a given movement class or pattern^4^, for example, the tennis serve. The “richness” of stored information in variable practice derives from four different sources: (a) previous conditions of execution (e.g., distances of a target), (b) response specifications (e.g., force and direction of movement produced), (c) sensory consequences of the produced response, and (d) effects of the movement on the environment. A greater variety of experience induced by variable practice of a movement class (e.g., tennis serve to different targets on the court), compared to constant practice (e.g., tennis serve to one target), produces a stronger schema that increases the transfer capability to a novel variation of the same movement class^5^. Note that the variability of practice hypothesis does not explain why variable random practice is more effective than variable blocked practice^3^.

Contextual interference studies compare the effects of variable random practice versus variable blocked practice in motor learning^6^. Two hypotheses based on memory processes were proposed. The elaborative-processing hypothesis proposed by Shea and Zimny^7^ claims that random practice involves a higher level of intratask and intertask comparisons between trials. Thus, the learner undergoes further elaboration and distinction in memory than in blocked practice. More elaborate information processing results in more comprehensive and readily retrievable memory traces. The forgetting-reconstruction hypothesis^8^, also named the action-plan reconstruction hypothesis, proposes that random practice promotes forgetfulness of a previously constructed action plan before each new trial. The learner has to constantly perform a different task during the next trial, and this process (involving the forgetting and reconstruction of action plans) strengthens the representation of these skills in memory. Conversely, during blocked practice, this process does not occur across repeated trials of the same task^8^. Both hypotheses do not distinguish if these benefits of random practice occur when variations of skills or variations of a same skill are required.

Recently, Lage *et al*.^3^ identified, through a review of the literature, ten articles that investigated the effects of practice organization at the neurophysiological level. The authors concluded that the results of the analyzed studies offer support to a greater cognitive effort during less repetitive practice. Cerebral areas directly associated with motor planning were most active during random practice than in blocked practice. It is important to emphasize that the purpose of these reviewed studies was to investigate the neural substrate involved in practice organization. There was no direct aim of investigating cognitive effort involved in different practice schedules. Moreover, the hemodynamic response observed via neuroimaging techniques does not directly translate to the level of cognitive effort of the learner in terms of information processing. There is no direct relationship between higher or lower cerebral activation and cognitive effort involved in the task performance. Additionally, there is a lack of temporal accuracy between neural changes with cognitive processing.

An interesting way to investigate the use of cognitive resources during task execution is the analysis of mental workload. Mental workload can be defined as a finite mental resource that one uses to perform a task under specific operational conditions^9^. Mental workload is affected by the demands of the task, which can be influenced by perceptual load, cognitive load and motor load^10,11^. If random practice demands a greater cognitive load as behavioral hypotheses have claimed, we could expect different levels of mental workload when comparing the same person engaged in a less repetitive order of execution and engaged in a more repetitive order of execution. Our first hypothesis is that random practice induces a greater mental workload than constant practice. To evaluate mental workload, we used two measures based on electroencephalography (EEG): EEG-engagement index and EEG-workload index. Both measures increase as a function of increasing task demands, but the EEG-engagement index tracks demands for sensory processing (e.g., information-gathering) and attention resources, while the EEG-workload index is a measure of the level of cognitive processes involving in working memory load, integration of information and problem-solving^12^.

The necessary level of mental workload required to perform a task is associated with the level of learning. Reduced mental workload is observed in advanced learning stages^13^. Novices, for example, present a higher mental workload in a determined task compared with more advanced performers^14^. The learning curve of errors in constant practice decreases faster and with higher magnitude than in random practice^15^, and these behaviors are associated with greater psychophysiological demand generated by random practice compared to constant practice^3^. Therefore, we could expect a decreasing level of mental workload in both EEG indexes during the motor practice independent of the practice schedule but with a more pronounced decline during constant rather than random practice. Thus, our second hypothesis is that mental workload will decrease more in constant practice than in random practice.

In summary, our objectives in the present study are (a) to investigate the level of mental workload involved in random and constant practice, and (b) to investigate the level of mental workload decreasing during constant and random practice. The mental workload is compared when a motor task is executed with three temporal variations and executed without temporal variations. Our hypotheses are (a) random practice induces greater mental workload in both EEG indexes than constant practice, and (b) mental workload in both EEG indexes will decrease more in constant practice than in random practice. The EEG approach not only has potential to evaluate these assumptions in a manner that behavioral methods have not yet revealed, but it also provides new explanations regarding the cognitive processes involved in less and more repetitive practice schedules.

## Results

### Relative error (RE)

**Figure 1:**
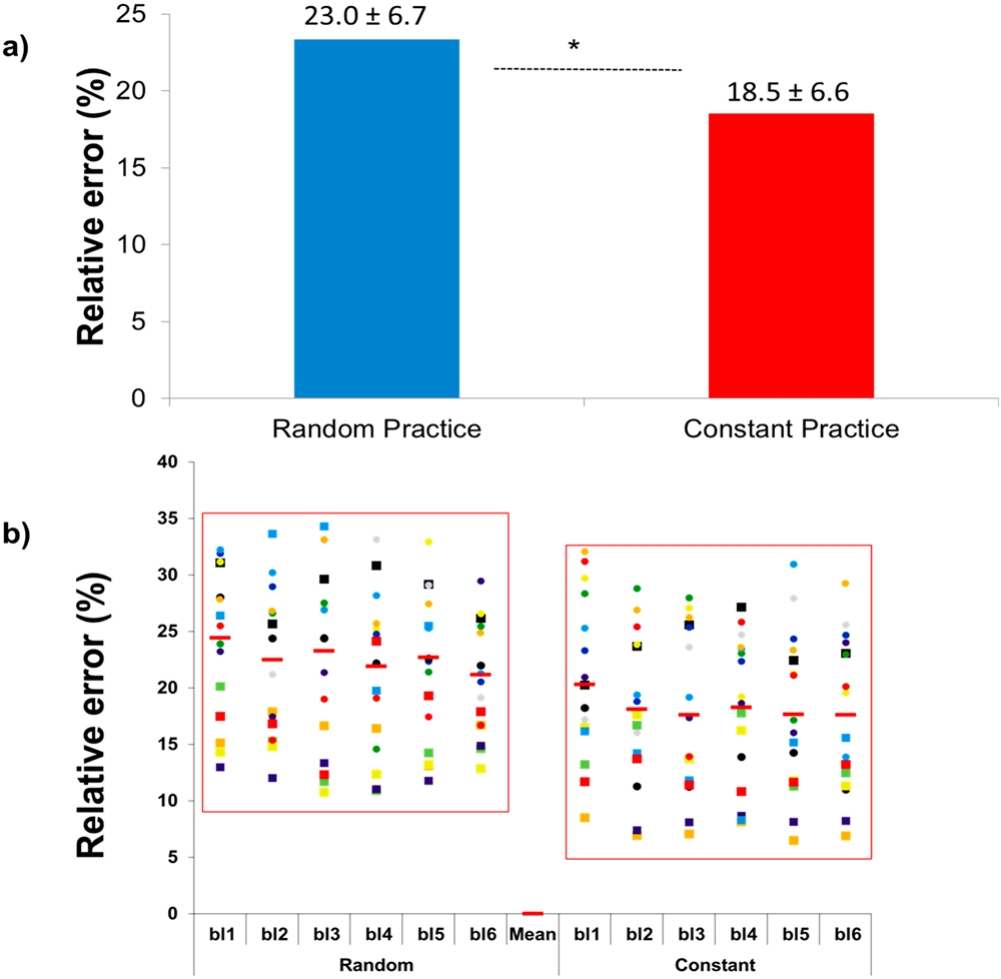
Relative error: (**a**) means of the relative error in the random and constant practice conditions, and (**b**) inter-subject variability over the blocks of trials (bl1…bl6) during both the random and constant practice conditions (*represents significant difference).

Descriptive statistics are presented in Fig. 1a and b. The paired *t*-test detected a difference between conditions [t(20) = −4.23, p < 0.01, d = 0.67]. The mean of relative error in random practice was higher than in constant practice.

### Absolute error (AE)

**Figure 2:**
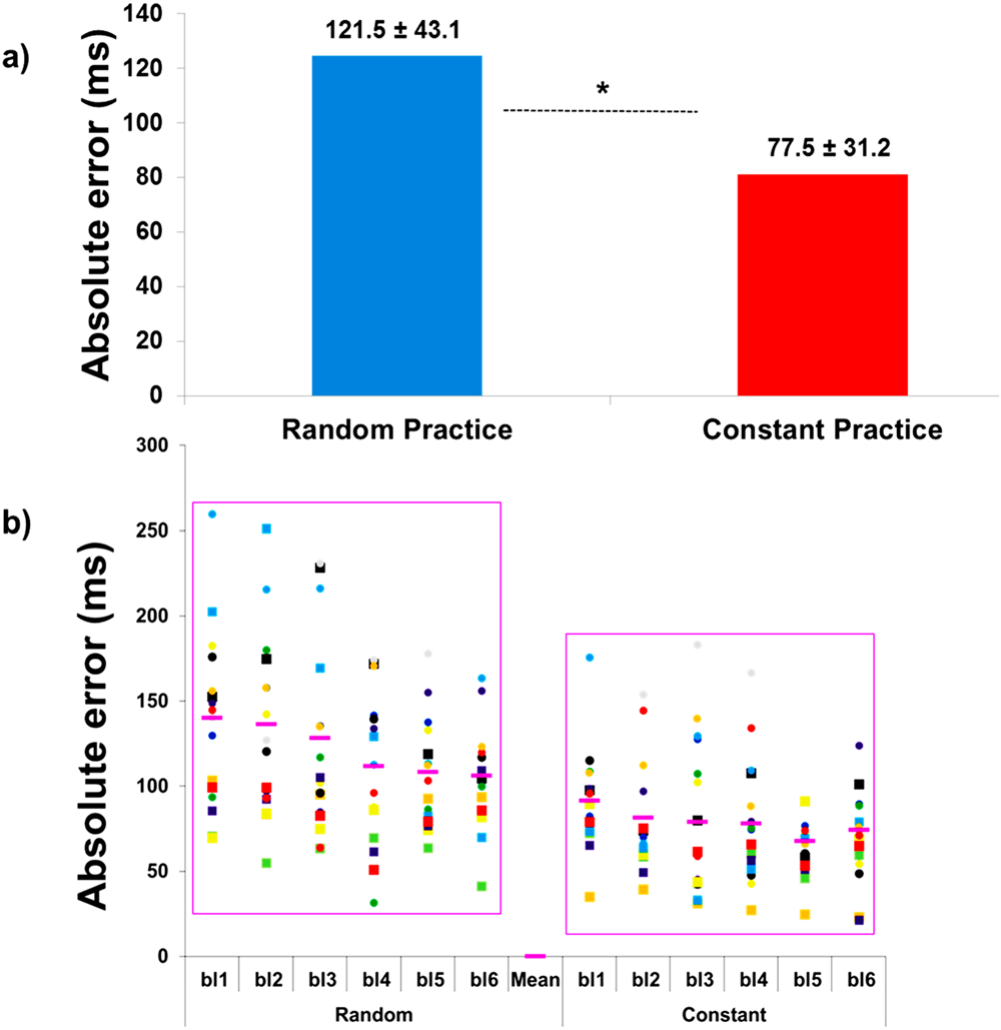
Absolute error: (**a**) means of the absolute error in the random and constant practice conditions, and (**b**) inter-subject variability over the blocks of trials (bl1…bl6) during both random and constant practice conditions (*represents significant difference).

Descriptive statistics are presented in Fig. 2a and b. The paired *t*-test detected a difference between conditions [t(20) = −6.61, p < 0.01, d = 1.16)]. The mean of absolute error in random practice was higher than in constant practice.

### EEG-engagement index

**Figure 3:**
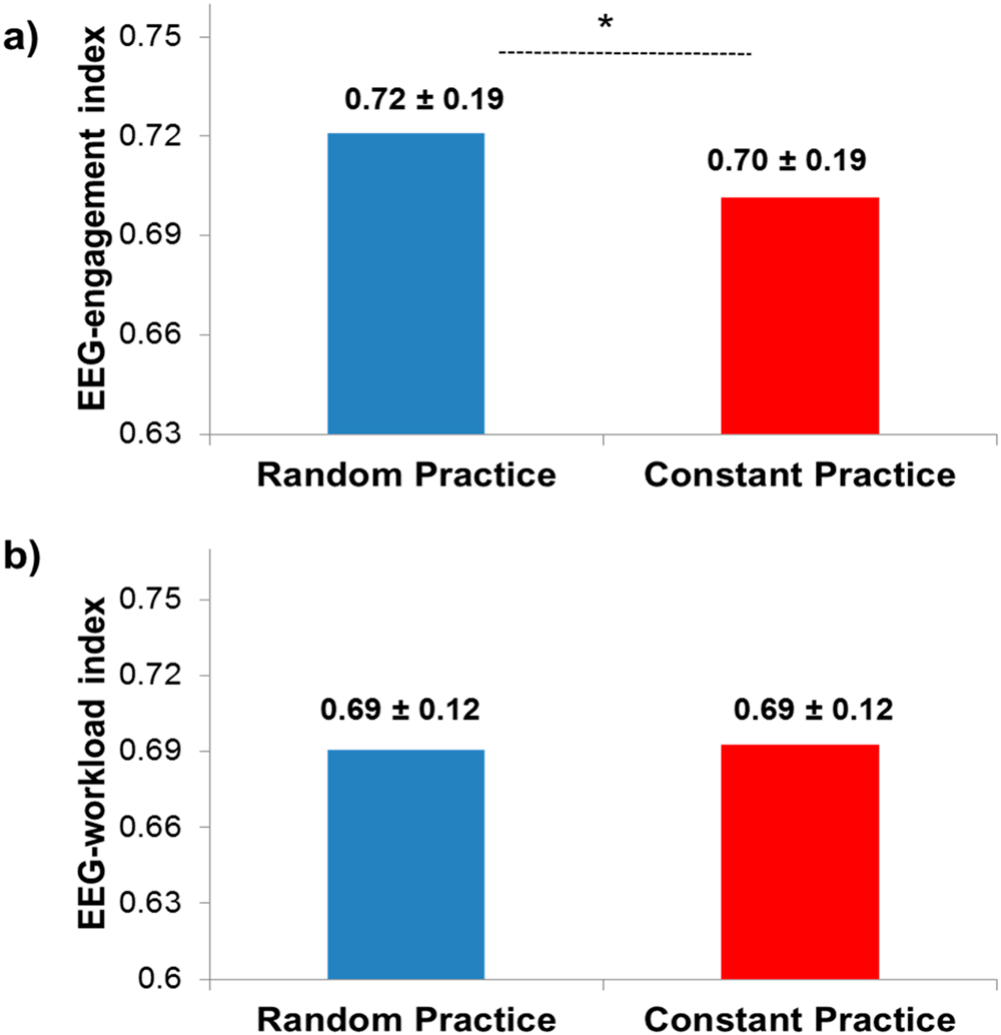
Means of the (**a)** EEG-engagement index and (**b**) EEG-workload index in the random and constant practice conditions (*represents significant difference).

Descriptive statistics are presented in Fig. 3a.The paired *t*-test indicated a significantly greater EEG-engagement index for the random practice condition compared to the constant condition [t(20) = −3.04, p < 0.01, d = 0.25], but in an analysis where order of conditions was included in the model, this result, while showing similar effect size, was no longer statistically significant (p > 0.05).

### EEG-workload index

Descriptive statistics are presented in Fig. 3b. The inferential analysis did not detect any difference between the random practice condition and the constant condition [t(20) = 0.33, p > 0.05, d = 0.03].

### EEG-engagement and EEG-workload index during the practice

**Figure 4:**
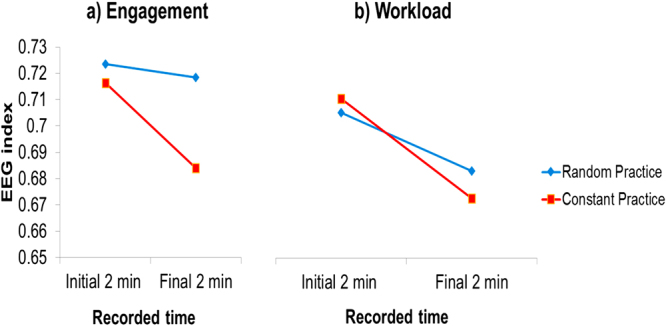
Means of the (**a)** EEG-engagement index and (**b**) EEG-workload index in the random and constant practice conditions during the initial and final two min.

**Figure 5:**
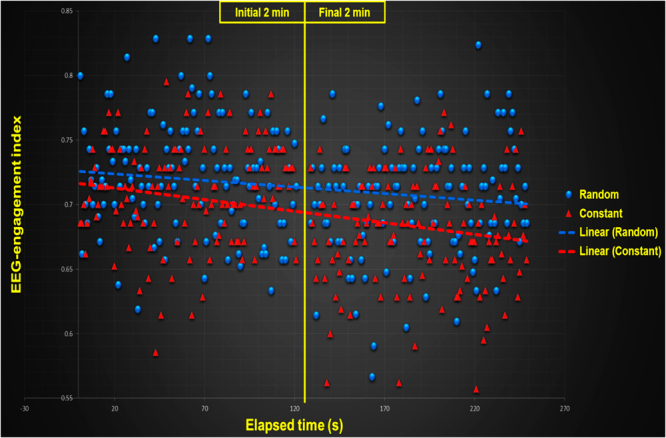
Dispersion of the EEG-engagement index means in each 1 second interval of the initial and final part of the constant and random practice conditions.

**Figure 6:**
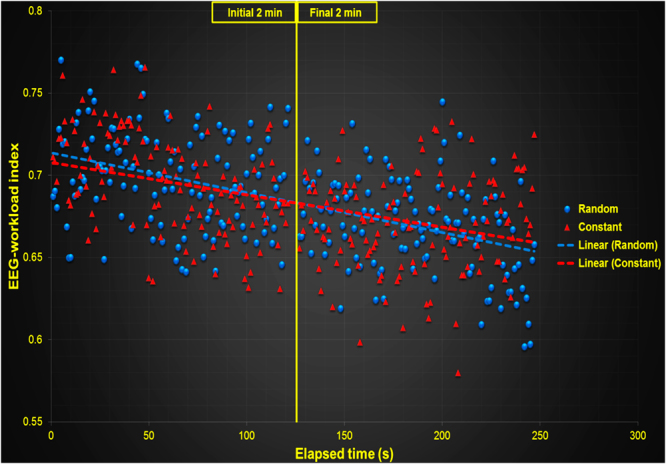
Dispersion of the EEG-workload index means in each 1-second interval of the initial and final part of the constant and random practice conditions.

The two-way ANOVA indicated a significant difference between the initial and final 2 min of the recorded time of subjects in both EEG-engagement, [F(3,20) = 8.917, p < 0.01, η^2^ = 0.30], and EEG-workload indexes, [F(3,20) = 4.195, p < 0.01, η² = 0.17]. The EEG-engagement index post hoc analysis indicated that (a) engagement diminished from the initial 2 min to the final 2 min in both constant and random conditions (p < 0.01, for both conditions), and (b) despite the fact that the engagement in the initial 2 min was identical between practice conditions, in the final 2 min, the random practice condition exhibited a greater level of engagement (p < 0.01). An analysis where order of conditions was included in the model showed significantly greater EEG-engagement index for the constant to random order in the final 2 min (p < 0.05). The EEG-workload index post hoc analysis indicated that workload diminished from the initial 2 min to the final 2 min only in the constant practice condition (p < 0.05). Figure 4 illustrates the mean of the subjects during the initial and final 2 min of EEG-engagement (Fig. 4a) and EEG-workload indexes (Fig. 4b). Both Figs 5 and 6 show the dispersion of means during each 1 second interval of the initial and final part of the constant and random practice conditions.

## Discussion

Overall, the results confirmed our hypotheses. Random practice induced greater cognitive effort than constant practice when the EEG-engagement index was analyzed. The analysis of the EEG-workload index did not show any differences between the practice conditions. However, throughout practice, EEG-workload index decreased significantly more in the constant practice condition than in the random practice condition. This effect was also observed in the EEG-engagement index. To our knowledge, all of these results are novel findings.

**Figure 7:**
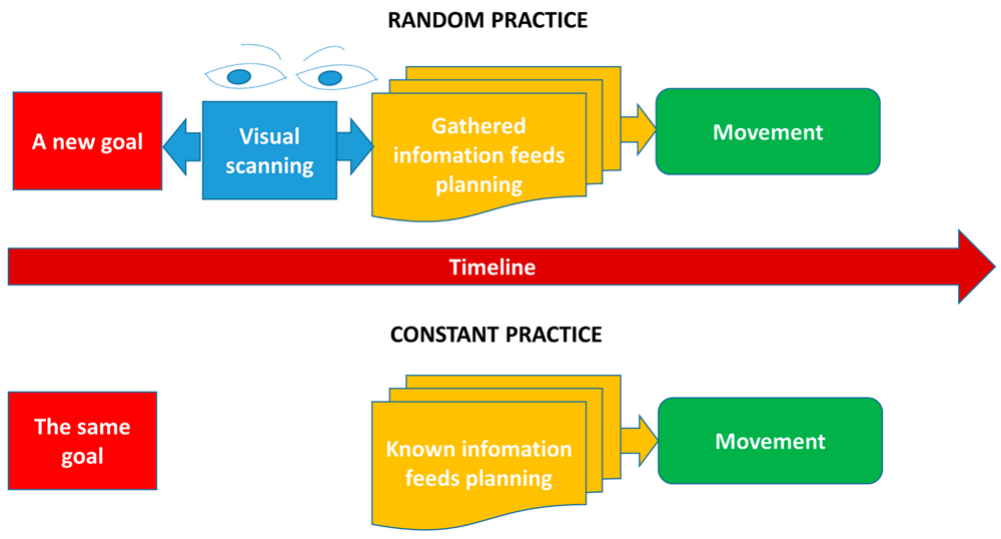
Process of visual scanning to gather information before movement planning. Random practice demand changes trial-by-trial in the absolute dimension requiring visual scanning from the learner to gather information about the new goal. In constant practice, the goal is the same trial-by-trial, diminishing the relevance of visual scanning and gathering information over the course of practice.

The explanatory hypotheses regarding the benefits of less repetitive practice schedules on motor learning emphasize the greater cognitive effort involving memory processes^4,7,8^. Interestingly, our results showed that difference between constant and random practice is more related with engagement than with working memory processes. Task engagement reflects the allocation of processing resources associated with information gathering, visual scanning, and sustained attention^16,17^. Despite the lack of discussion regarding sensory processing in the practice organization literature, increased demand for sensory processing would be expected in random practice. The learner needs to address the continuous changes imposed trial-by-trial in the absolute dimension and the execution of a stable relative dimension of the task. In each new trial, the learner needs to perform visual scanning to gather information about which new parameter value (total movement time) is required for the first stage of movement planning (Fig. 7).

The relevance of the process of visual scanning to gather information before movement planning should decrease rapidly in constant practice. The trial-to-trial stability promoted by the consecutive repetition of the relative and absolute dimensions does not require the learner to search for previous information about movement’s goals to supply the next planning step. The learner knows what is required of him or her (Fig. 7). The greater EEG-engagement index found for the constant to random order in the final 2 min reinforce this hypothesis. Curiously, this possible difference in information processing when one practices in a random or constant order has not been discussed in the literature of practice organization. We can extend this hypothesis to blocked practice, which shares similarities with constant practice in terms of consecutive repetition of the same movement.

This difference in the importance of visual scanning and information gathering between constant and random practice schedules should be increased in a more complex task, such as playing a sport. If we use the volleyball serve as an example, the increased necessity of analysis of the previous conditions of execution during random practice imposes a detailed visual search to gather information. The learner needs to evaluate the relationships between (a) position and the distance of the new target positioned on the other side of the court, (b) his or her body position on the court, and (c) the height of the net, among other variables.

The visual scanning and information gathering after the movement execution seem, at first sight, to be the same between the practice conditions. Once the movement is finished, two main sources of information are accessed. First, information provided by intrinsic feedback is gathered, and then the extrinsic information (knowledge of results or KR) about the relative and absolute dimensions is gathered. In both constant and random conditions of practice, the learner needs to gather these types of information. However, a possible difference is the time or frequency of visual fixation spent in KR information related to the absolute and relative dimensions. The learner in the random practice condition seems to direct his or her attention more to the leaning of the absolute dimension of the task, because the performer is concerned with constantly changing in the absolute dimension^8^. Conversely, during constant practice, attention is focused more in the relative dimension of the task. Supporting this view, it is well known that constant practice benefits learning the relative timing segments of the movement^15,18^. The positive effect of random practice on the learning movement parameters is also well known^18–20^. In a pilot study, Lage *et al*.^21^ observed a significantly higher frequency of visual fixations on the absolute error (obtained by KR) during random practice than in constant practice. Overall, all hypotheses here presented on differences in visual scanning and information gathering promoted by constant and random practice before and after movement can be further investigated through analyses of oculomotor behavior.

When we analyzed the results of the EEG-workload index, the means observed in the constant and random practice were similar. This index is a measure of cognitive effort considered more the domain of executive function, such as working memory and reasoning^12^. Executive control involving working memory requires the active maintenance and manipulation of a particular type of information^22,23^. Some functions of working memory, such as translating instructions into action plans, integrating new information into the action plans (updating), and perceiving relations between items^24^ seem to be more requested in random practice than in constant practice. In fact, the explanatory hypotheses regarding the benefits of less repetitive practice schedules have described in some degree the depth of these functions^7,8^.

Assuming that reasoning would be not possible without working memory^24^ or that reasoning is nothing more than working memory capacity^25^, one could expect that the high demand on working memory in random practice is also associated with higher levels of reasoning involved in this type of practice. It is also a relationship not discussed in the organization of practice literature. If working memory and reasoning are higher in random practice, why is the index of EEG-workload equal between random and constant conditions? Errors in learning curves during constant practice decrease faster and in higher magnitude than in random practice^15^. The initial part of the practice, when the level of error is high, seems to require a high level of executive control, not only for random practice but also for constant practice. Over the course of practice, the repetitive characteristic of constant practice diminishes the necessity of translating instructions into action plans, integrating new information into the action plans (updating), and perceiving relationships between items. Conversely, in random practice, all of these processes are maintained in high demand throughout practice. Thus, the difference in the EEG-workload index is better observed when isolating the initial and final moment of practice.

Berka *et al*.^12^ observed that a higher number of items to be encoded in the backward digit span test increases the EEG-workload index. In constant practice, the translating of goal into an action plan is quickly encoded. The degree of adjustments in each new trial diminish because KR regarding the last trial is easily integrated into the action plan, different from random practice, in which the KR is not related with the next action, requiring relationships between the present trial and information from past trials. When observing the less repetitive random practice condition, we can hypothesize that the decay rate on the demand of these processes is less than in constant practice.

Compared to the EEG-workload index, the level of engagement exhibited a decrement from the initial to the last part of the practice for both constant and random conditions. These results indicate that EEG-workload is maintained at a high level in random practice, but engagement is affected by practice. The processes of visual scanning and information gathering are also modified trial-by-trial in random practice. However, in the last trials, the level of engagement decayed more in constant practice than in random practice. These results reinforce our hypothesis that the relevance of the process of visual scanning to gather information before movement planning should decrease more in constant practice than in random practice. The initial level of engagement was identical between conditions but different in the final trial of practice.

Overall, the results showed that cognitive effort involved in random practice is greater, not only in memory processes, as discussed in the behavioral studies, but also in sensory processes. The level of difficulty generated by the non-consecutive repetition of task parameters reflects important cognitive processes associated with motor control and learning. A limitation of this study was to recruit only males. This choice diminish the generality of our findings. Studies in which sex is counterbalanced are recommended. Future studies should attempt to investigate the relationship between mental workload and performance observed during learning tests, such as retention and transfer tests. This proposition is a challenge because mental workload is easily analyzed intra-subject, not inter-subject, as usually applied in motor learning studies with different groups of practice. Another perspective is to evaluate the time-locked mental workload during planning, movement execution and KR evaluation. This type of approach has the potential to better explain the differences between the level of mental workload involved in sensory processes and working memory processes.

## Methods

### Participants

Twenty-two men, all undergraduate students ranging from 18 to 35 years of age, participated in this study. One participant was excluded because EEG data was missing due to a technical failure. Therefore, the final sample consisted of 21 participants (mean 24.09 ± 4.04). All participants were right-handed university students (mean laterality quotient = 86.1 in the Edinburgh Handedness Inventory^26^, and had normal or corrected-to-normal visual acuity in both eyes. All volunteers had no prior experience with the motor task.

### Ethical statement

All methods were carried out in accordance with the Helsinki Declaration. Ethical approval for the study was granted by the Ethics Committee of the Federal University of Viçosa. All participants provided informed consent to participate as volunteers.

### Apparatus

The B-Alert × 10 sensor headset (Advanced Brain Monitoring Inc., Carlsbad, CA, USA) was used to acquire the electroencephalography results (EEG). Nine Ag/AgCl EEG electrodes were located at F3, Fz, F4, C3, Cz, C4, P3, POz, and P4, according to the international 10–20 system. Bi-polar recordings (F3-F4, C3-C4, Cz-PO, F3-Cz, Fz-C3, Fz-PO) were selected in order to reduce the potential for artifacts generated by movements. Two electrodes on the mastoid bones (left and right) were used as the reference and ground. The sampling rate was 256 samples/s for all channels and transferred in real-time via Bluetooth link to a host computer where the B-Alert software (Advanced Brain Monitoring Inc., Carlsbad, CA) stored and processed the EEG data. B-Alert software classifies the level of task engagement into one of four levels of alertness (sleep onset, distraction, low engagement and high engagement) and the level of mental workload.

A computer, color monitor, and numeric keypad were placed on a standard table. A custom-made software program was used to control the motor task and to register the time between pressing the keys^27,28^. Participants were asked to sit on a chair in front of the computer monitor and to adjust the numeric keypad position to comfortably use it with their right hand.

### Motor task

**Figure 8:**
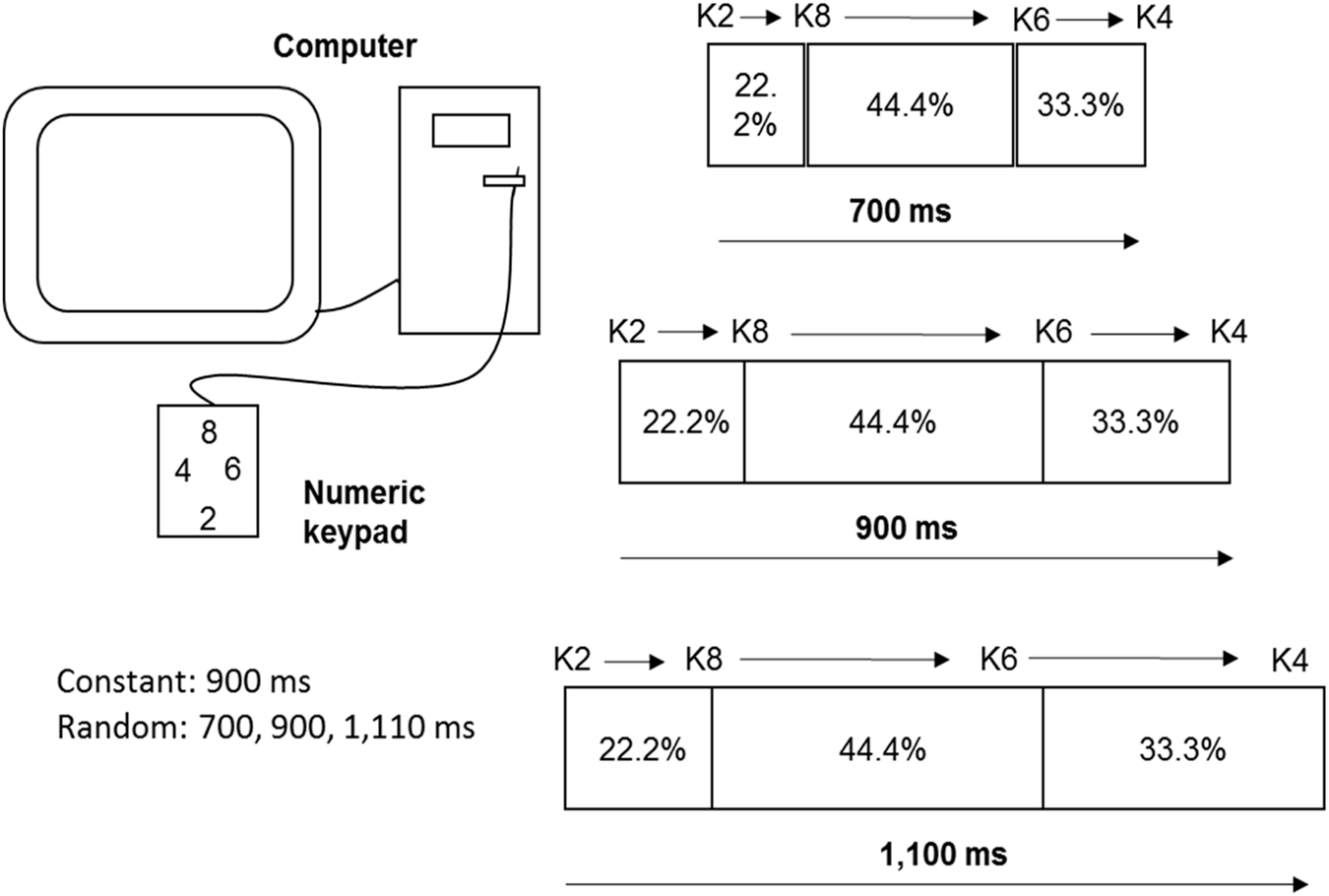
Apparatus used to apply the motor task, the sequence of keys typed (K2…K8), the relative criterion segment ratios between keys (22.2%, 44.4%, and 33.3%) and the total criterion movement times (700, 900, and 1,100 ms).

The participants were asked to sequentially press four keys (2, 8, 6, and 4) on the numeric keypad. The task was performed with the index finger of their right hand during all phases of the experiment. The total criterion movement time (MT) and the relative criterion segment ratios were presented on the computer screen before each trial. The MT was 900 ms for the constant practice condition and 700, 900, and 1,100 ms for the random practice condition. Relative to the MT, the criterion segment ratios were 22.2% (key 2 to 8), 44.4% (key 8 to 6) and 33.3% (key 6 to 4) in all experimental phases (Fig. 8). After completing each trial, the knowledge of the results was displayed on the screen. The knowledge of the results included the total movement time performed, the relative criterion segment ratios of the total criterion time for each segment, the relative error (RE) of the sum of the criterion segment ratios.

### Procedures

First, participants filled out the Edinburgh Handedness Inventory^26^. Then, the EEG headset was placed onto the head of the volunteer. Subsequently, the EEG device was connected, the communication port was activated, and the impedance was evaluated via B-Alert software. Monitoring was conducted to evaluate the quality of data acquisition. Finally, data acquisition began. The acquisition of baseline data, named Metric Benchmark, was used to create the individualized EEG profiles required for the index of cognitive states (engagement and workload metrics) to be valid and accurate across individuals. Each participant complete benchmarking session (15 min) included three distinct tasks: 3-choice vigilance task; a Visual Psychomotor Vigilance Task; and an Auditory Psychomotor Vigilance Task^29^.

After the benchmarking session, instructions for the motor task were provided to each volunteer. The volunteer was asked to be as accurate as possible concerning both the MT and relative criteria. Before each trial, the MT and the criterion segment ratios for the task were displayed on the screen. After the movement sequence was completed, the results were presented for 6 seconds.

The study consisted of two conditions: the random practice condition and the constant practice condition. The order of conditions was counterbalanced across participants. Eleven participants performed constant practice followed by random practice and ten participants practiced in a contrary order. In each condition, participants performed 60 trials with EEG data acquisition. The EEG was recorded separately in each practice condition (maximum recording time = 10 min). During constant practice, participants performed the same MT (900 ms) and criterion segment ratios (22%, 44% and 33%). In random practice, participants randomly performed three MTs (700, 900 and 1,100 ms) and the same criterion segment ratios (22%, 44% and 33%). Participants were counterbalanced by order of the initial practice.

### EGG signal processing and data reduction

All signal processing consisted of (a) filtering and digitization, (b) artifact identification and decontamination, and (c) feature extraction. These processing steps are performed automatically by the B-Alert system and have been previously published^12,30^. To remove environmental artifacts, a Notch filter at 60 Hz is applied to all EEG data. Spikes, excursions, and amplifier saturation related to movement are analyzed in the time domain. Amplifier saturation is identified when the amplitude between two data points exceeded predefined thresholds (e.g., 440 µV) or the EEG amplitude approached the maximum or minimum of the amplifier dynamic range. Spikes and excursions are automatically identified when the EEG amplitude exceed 40 µV over short durations (e.g., between 12–27 ms). Afterwards, the EEG is deconstructed using a wavelet transformation into the 0–2, 2–4, 4–8, 8–16, 16–32, 32–64, and 64–128 Hz wavelet bands. Wavelet power in the 64–128 Hz band identified and excluded excessive muscle activity¹².

Eye blinks are detected by the linear discriminant function analysis. The absolute value of the wavelet coefficients (0–2, 2–4, 4–8, 8–16, and 16–32 Hz) from the data points (50th, 40th 30th, 20th, and 10th) before and after the target data point from FzPOz and CzPOz are used as variables to classify each data point as an eye blink, theta wave, or non-eye blink. The eye blink region is identified by multiple data points classified as eye blinks. The eye blink region is defined through a fixed distance before the start (e.g., 50 data points) and after the end (e.g., 50 data points) of the eye blink. Decontamination is accomplished by computing mean wavelet coefficients for the 0–2, 2–4, and 4–8 Hz bins from nearby non-contaminated regions and replacing the contaminated data points. Then, wavelet bands (except 64–128 Hz) are used to reconstruct the EEG signal. The data points previously associated with excursions, spikes and saturation are replaced with zero values at zero crossing before and after excursions, spikes and saturations. Lastly, EEG absolute and relative power spectral density (PSD) variables for each 1-s epoch are computed running a Fast-Fourier transform applied using a 50% overlapping Kaiser window (α = 6.0). The PSD values are then scaled to accommodate the insertion of zero values as replacements for the artifact^12^.

To obtain the EEG-cognitive metrics, a four-class quadratic discriminant function analysis (DFA) was derived for each participant. The model was individualized for each participant using DFA coefficients derived during Metric Benchmark. A linear DFA with two classes was used to obtain the EEG-workload metrics (see detailed explanation in^12^.

### Measurements and data analyses

Two dependent motor variables were measured: (1) the relative error (RE) and (2) the absolute error (AE). The RE was used as a measure of proficiency in the relative dimension, while the AE was used as a measure of proficiency in the absolute dimension. The RE was determined as the sum of absolute differences between the observed and criterion time ratios for each segment computed as follows:

$${\rm{RE}}={\rm{{\rm I}}}{\rm{R}}1-22.2{\rm{{\rm I}}}+{\rm{{\rm I}}}{\rm{R}}2-44.4{\rm{{\rm I}}}+{\rm{{\rm I}}}{\rm{R}}3-33.3{\rm{{\rm I}}}$$

where Rn = (the actual movement time of segment/total movement time) × 100.

The AE was computed as the difference between the actual movement time and the total criterion time:

$${\rm{AE}}=({\rm{MTn}}-{\rm{total}}\,{\rm{criterion}}\,{\rm{time}}).$$

The means of ER and EA from the 60 trials in the constant and random conditions were calculated. The Shapiro-Wilk Test showed a normal distribution for the two indexes. Thus, a Student’s *t* test for dependent samples for groups was conducted. The effect size was calculated using Cohen’s formula^31^. These analyses were conducted to confirm the higher level of ER and EA expected in random practice compared to constant practice.

To evaluate the degree of cognitive effort required by constant and random practice schedules, two EEG-based measures of cognitive states were used: (a) task engagement defined as EEG-engagement index and (b) mental workload defined as EEG-workload index. Both indexes increase as a function of increasing task demands. However, the EEG-engagement index tracks demands for sensory processing and attention resources, while the EEG-workload index is a measure of the level of cognitive processes involving working memory load, integration of information and problem-solving^12^.

Means of the participant’s EEG-engagement and EEG-workload indexes obtained during the EEG record time were calculated. The Shapiro-Wilk Test showed a normal distribution for the two indexes. Thus, a Student’s *t* test for dependent samples for groups was conducted. The effect size was calculated using Cohen’s formula^31^.These analyses were conducted to investigate the level of mental workload involved in random and constant practice.

The means of the two indexes were also computed from the initial 2 min and the final 2 min of the record time of each participant. These two means were used to investigate the level of mental workload decreasing during constant and random practice. The Shapiro-Wilk Test showed a normal distribution for the two indexes in the two moments of the practice. Thus, a Two-way ANOVA was conducted for both EEG-engagement and EEG-workload indexes. The effect size was calculated using eta-squared (ƞ²). These analyses were applied to investigate the level of mental workload decreasing during the practice.

A significant difference at the level of *α* = 0.05 was adopted for all statistical analyses.

### Data availability statement

The authors declare that all data are available.
