# Author Correction: Task engagement and mental workload involved in variation and repetition of a motor skill

**DOI:** 10.1038/s41598-021-89268-3

**Published:** 2021-05-11

**Authors:** Natália Lelis-Torres, Herbert Ugrinowitsch, Tércio Apolinário-Souza, Rodolfo N. Benda, Guilherme M. Lage

**Affiliations:** School of Physical Education, Physiotherapy and Occupational Therapy, Universidade Federal de Minas Gerais, Av. Presidente Carlos Luz, 6627, Pampulha, Belo Horizonte, 31270-901 Brazil

Correction to: *Scientific Reports* 10.1038/s41598-017-15343-3, published online 07 November 2017

The original version of this Article contained errors.

The standard deviation values shown in Figure 1a, Figure 2a and Figure 3a,b were incorrectly given.

The original Figures 1–3 and accompanying figure legends appear below.

**Figure 1:**
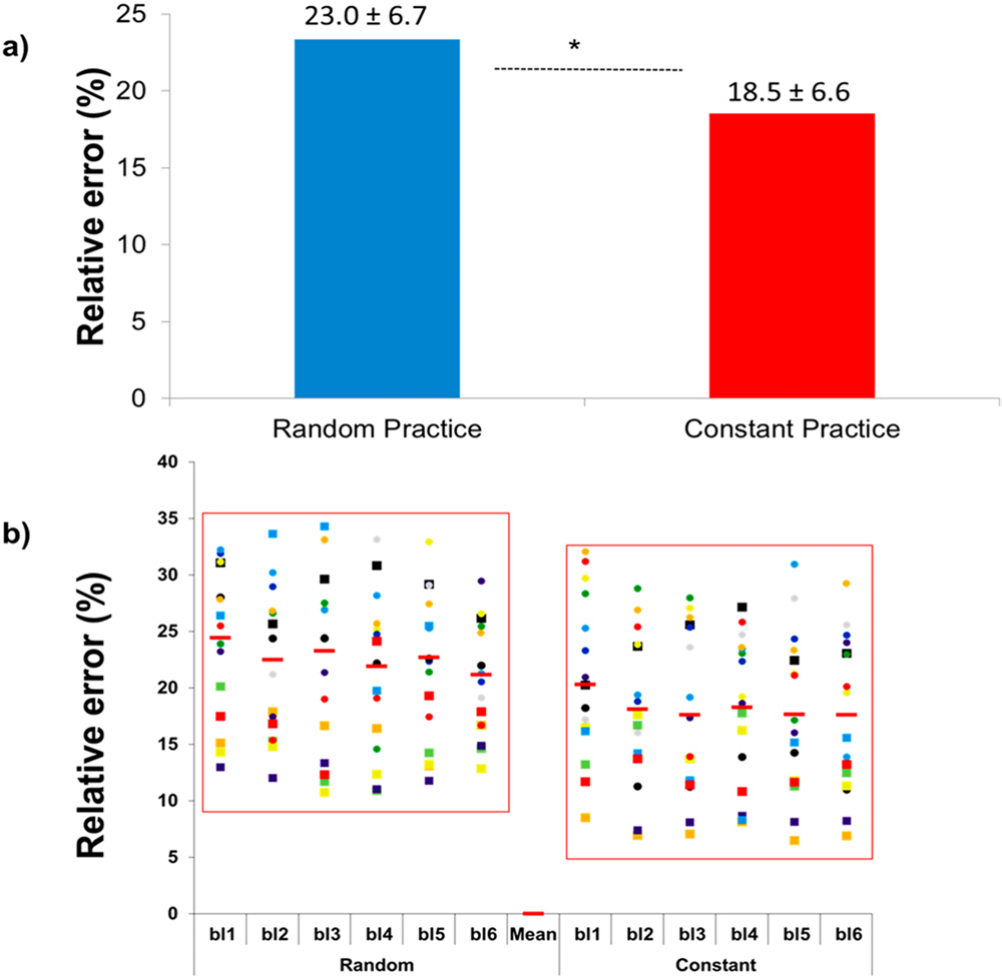
Relative error: (**a**) means of the relative error in the random and constant practice conditions, and (**b**) inter-subject variability over the blocks of trials (bl1…bl6) during both the random and constant practice conditions (*represents significant difference).

**Figure 2:**
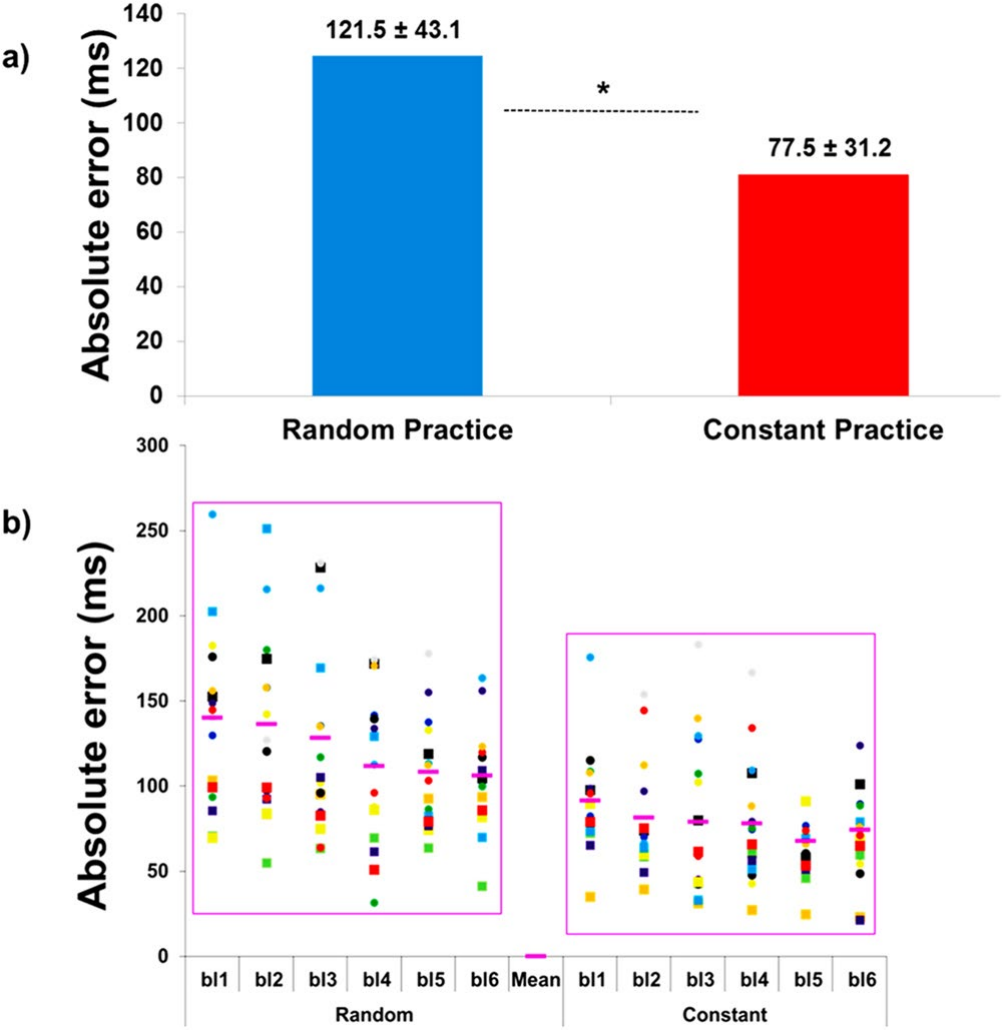
Absolute error: (**a**) means of the absolute error in the random and constant practice conditions, and (**b**) inter-subject variability over the blocks of trials (bl1…bl6) during both random and constant practice conditions (*represents significant difference).

**Figure 3:**
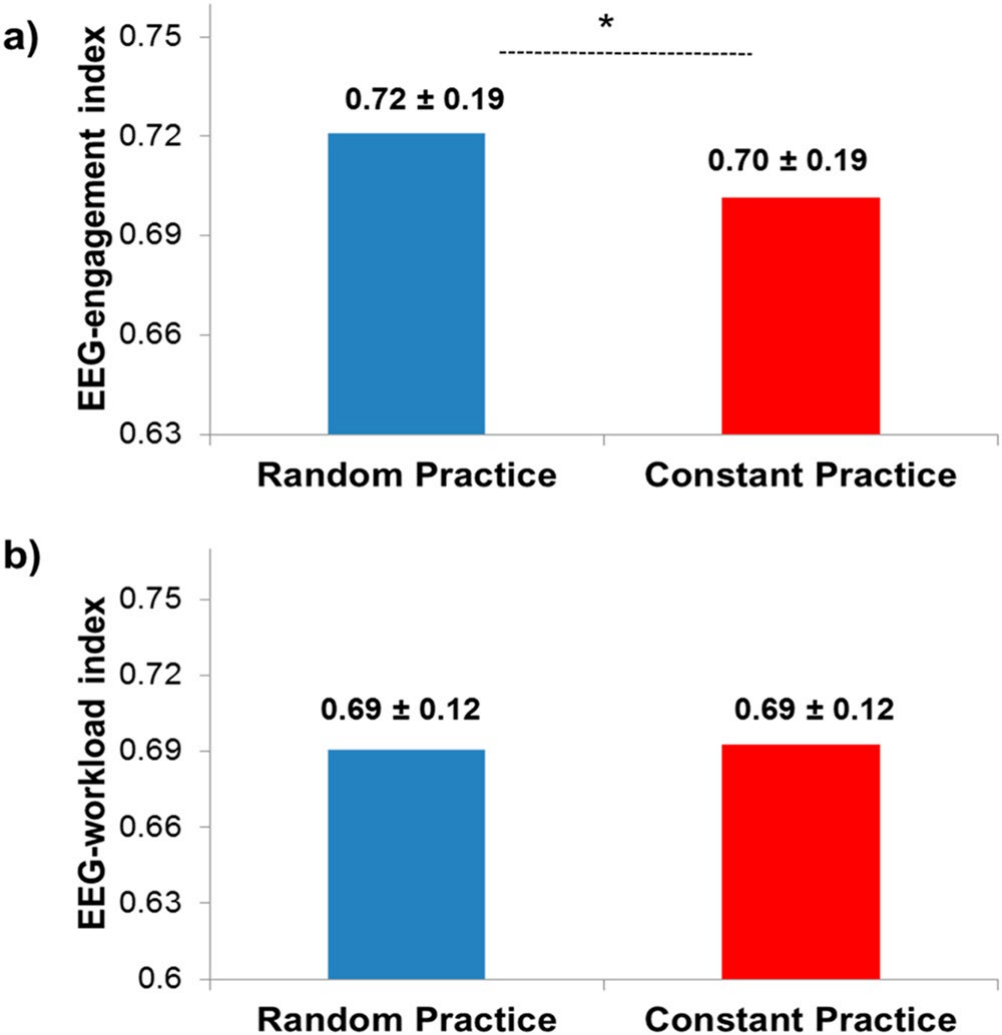
Means of the (**a)** EEG-engagement index and (**b**) EEG-workload index in the random and constant practice conditions (*represents significant difference).

The original Article has been corrected.

